# Treatment of Habitual Constipation

**Published:** 1851-03

**Authors:** J. C. Warren


*Treatment of Habitual Constipation.* By Dr. J. C. Warren.—The treatment of chronic costiveness, an ailment so common in persons who lead sedentary lives, may be either medicinal or alimentary. Of medicines, Dr. Warren employs but three, aloes, magnesia, and senna-wine. He considers that the bitter principle of *aloes* renders it in some measure a substitute for the deficient bile, and a few grains of the powder or gum forms a mild and effectual aperient. The watery extract (5 to 10 grains), combined with an aromatic oil, is one of the mildest of laxatives, taken fasting. Aperients should not be taken at night, when they become buried in the alimentary mass, and ineffectual, or in their action disturb the repose of patients of an irritable temperament; but when the stomach is empty, either before dinner or breakfast. *Magnesia*, however, is so mild an aperient, that even if taken at night, it does not disturb sleep, and, from its combining with the acids which have been generated during the day, is then even more likely to be useful. When the stomach is not acid, half an orange aids its action. *Senna-wine*, prepared according to Dr. Lane's process, is a very satisfactory aperient, the dose being from ½oz. to 2oz., and as it is slightly stimulant, it may be taken for an oppressed stomach at night. If taken in the morning it should be diluted with equal parts of water.

Among the *alimentary* substances which tend to relieve constipation in some persons, fruit is the most agreeable; but for this end it should be taken before meals, when the stomach is empty—its employment after a full meal often giving rise to acetous fermentation and consequent disorders. Laxative vegetables are not usually easily digested by persons of weak stomachs, in whom they cause distressing flatulence. In other subjects they may act very beneficially. The costiveness which occurs after weaning is removed by a slightly sweetened infusion of cranberries. Some persons find advantage in drinking a glass of cold water, others a cup of strong coffee, early in the morning. Animal food is rather laxative than not, especially the fatty portions which, however, delicate stomachs cannot manage. Some people take much wine, believing it acts on the bowels. If it does so, it is from the mere weight of the fluid, or perhaps from its causing a toxical effect, when the bowels take on a conservative eliminatory action. Most wines and spirits, taken in moderate quantities, adstrict the bowels.

Fine flour is highly nutritious; and if exclusively used, like other nutritious substances, as jelly, arrow-root, milk, etc., causes costiveness. The artificial separation of the covering of the corn is counteractive of the intentions of nature, this stimulating the intestine to expulsive action. After employing, with great advantage, bread containing the *bran* in his own family, and recommending it to numbers of others, the author was induced to try a still coarser preparation. Wheat was ground in a coffee-mill, and then boiled with a succession of water and a little salt, for three or four hours. This Dr. Warren has found incalculably *the best and pleasantest remedy for constipation*, effecting quite a revolution in the economy and health, when taken in sufficient quantites (12oz.), either as a part or whole of the breakfast, or instead of pudding and vegetables at dinner. When the stomach will bear sweet substances, honey, molasses, etc., may be added with advantage. A moderate degree of fluidity, i. e., less than that of boiled rice or hominy, increases the laxative power. The wheat acts in part by its mere bulk, and probably in part by reason of the stimulating effect of the particles of bran.—*Am. Jour. Med. Sci.*

